# ICU Blood Pressure Variability May Predict Nadir of Respiratory Depression After Coronary Artery Bypass Surgery

**DOI:** 10.3389/fnins.2015.00506

**Published:** 2016-01-11

**Authors:** Anne S. M. Costa, Paulo H. M. Costa, Carlos E. B. de Lima, Luiz E. M. Pádua, Luciana A. Campos, Ovidiu C. Baltatu

**Affiliations:** ^1^Center of Innovation, Technology and Education, Camilo Castelo Branco UniversitySao Jose dos Campos, Brazil; ^2^Health Sciences Center, State University of PiauiTeresina, Brazil; ^3^Hospital Sao MarcosTeresina, Brazil; ^4^Health Sciences Center, Federal University of PiauiTeresina, Brazil

**Keywords:** CABG, blood pressure variability, ICU, autonomic nervous system, respiratory function, perioperative parameters

## Abstract

**Objectives:** Surgical stress induces alterations on sympathovagal balance that can be determined through assessment of blood pressure variability. Coronary artery bypass graft surgery (CABG) is associated with postoperative respiratory depression. In this study we aimed at investigating ICU blood pressure variability and other perioperative parameters that could predict the nadir of postoperative respiratory function impairment.

**Methods:** This prospective observational study evaluated 44 coronary artery disease patients subjected to coronary artery bypass surgery (CABG) with cardiopulmonary bypass (CPB). At the ICU, mean arterial pressure (MAP) was monitored every 30 min for 3 days. MAP variability was evaluated through: standard deviation (SD), coefficient of variation (CV), variation independent of mean (VIM), and average successive variability (ASV). Respiratory function was assessed through maximal inspiratory (MIP) and expiratory (MEP) pressures and peak expiratory flow (PEF) determined 1 day before surgery and on the postoperative days 3rd to 7th. Intraoperative parameters (volume of cardioplegia, CPB duration, aortic cross-clamp time, number of grafts) were also monitored.

**Results:** Since, we aimed at studying patients without confounding effects of postoperative complications on respiratory function, we had enrolled a cohort of low risk EuroSCORE (European System for Cardiac Operative Risk Evaluation) with < 2. Respiratory parameters MIP, MEP, and PEF were significantly depressed for 4–5 days postoperatively. Of all MAP variability parameters, the ASV had a significant good positive Spearman correlation (rho coefficients ranging from 0.45 to 0.65, *p* < 0.01) with the 3-day nadir of PEF after cardiac surgery. Also, CV and VIM of MAP were significantly associated with nadir days of MEP and PEF. None of the intraoperative parameters had any correlation with the postoperative respiratory depression.

**Conclusions:** Variability parameters ASV, CV, and VIM of the MAP monitored at ICU may have predictive value for the depression of respiratory function after cardiac surgery as determined by peak expiratory flow and maximal expiratory pressure.

**ClinicalTrials.gov Identifier:** NCT02074371.

## Introduction

Blood pressure variability reflects different physiological phenomena and represents a subject of actual investigation as potential prognostic value and risk stratification (Boggia et al., 2014). Novel indices of blood pressure variability, as assessed by 24-h ambulatory monitoring have been proposed to provide a more accurate and elaborate estimation of cardiovascular risk (Campos et al., 2013). Blood pressure variability is controlled by neurohumoral factors and it has frequently been used as marker of autonomic tone and sympathovagal balance (Charkoudian and Wallin, 2014).

Surgical stress induces alterations of blood pressure variability as result of neurohumoral and autonomic reactions to stress (Souza Neto et al., 2004). Perioperative blood pressure variability has been associated with 30-day mortality after cardiac surgery (Aronson et al., 2011), possible mechanisms including autonomic imbalance (Pantoni et al., 2014), sympathetic overdrive, and parasympathetic withdrawal (Patron et al., 2014; Ksela et al., 2015).

Autonomic system controls both cardiovascular and respiratory systems through a single neural system conceiving a reciprocal interaction between the respiratory and autonomic control. This reciprocal interaction between the autonomic cardiovascular and respiratory control systems postulates the principle of cardiorespiratory coupling (Dick et al., 2014). Alterations in the cardiorespiratory coupling appear after surgery (Murray et al., 2009; Politano et al., 2013). In fact, among the most serious postoperative adverse events commonly occurring in cardiac surgery is respiratory depression, which can potentially lead to subsequent pulmonary complications and death (Pompei and Della Rocca, 2013). Such pulmonary complications include alveolar atelectasis, acute lung injury/pulmonary edema, acute cardiogenic pulmonary edema (CPE), pulmonary embolism or infection (Neves et al., 2013).

In this study, we hypothesized that the blood pressure variability at the post surgery intensive care unit (ICU) that may reflect the perioperative neurohumoral challenges and autonomic alterations of cardiorespiratory coupling could further predict the respiratory depression occurring after the postoperative ICU period. Postoperative respiratory depression was investigated after coronary artery bypass graft (CABG) since it has been described as a decrease in respiratory pressures (maximum inspiratory pressure—MIP and maximum expiratory pressure—MEP) and peak expiratory flow (PEF) in comparison to preoperative values (Westerdahl et al., 2005; Stein et al., 2009; Hirschhorn et al., 2012). The primary endpoint of this study was to investigate whether the blood pressure variability in the intensive care unit (ICU) is correlated with the nadir of postoperative respiratory function impairment after CABG with cardiopulmonary bypass (CPB).

## Methods

A prospective observational study was conducted on 44 patients with coronary artery disease submitted to elective CABG with CPB at Hospital Sao Marcos, Teresina, Brazil. Data were collected preoperatively, intraoperatively and up to the 7th postoperative day.

The study was approved by the Research Ethics Committee of the Faculty of Medical Sciences, State University of Piauí, and by the Ethics Committee of Hospital São Marcos, in accordance with Resolution 466/12 of the National Health Council (Ministry of Health) for research on human beings (Permit No. 128/11). Written informed consent was obtained from all patients before enrollment.

The CABG surgery and perioperative medication and monitoring management were standardized according with the 2011 ACCF/AHA Guideline for Coronary Artery Bypass Graft Surgery (Hillis et al., 2011).

The patient inclusion criteria were: patients of both sexes older than 18 years of age submitted to CABG with CPB with coronary disease confirmed by coronary angiography, with no chronic or acute pulmonary disease, with bypass graft of the left internal thoracic artery and/or saphena, who remained in spontaneous ventilation on the first postoperative day, discharged from ICU in the 3rd day followed by 7 days stay at the postoperative unit, and with an EuroSCORE (European System for Cardiac Operative Risk Evaluation) of < 2. EuroSCORE is a well-established method of calculating predicted operative mortality for patients undergoing cardiac surgery. It includes patient-related factors (age, sex, chronic pulmonary disease, extracardiac arteriopathy, neurological dysfunction disease, previous cardiac surgery, serum creatinine over 200 μmol/l, active endocarditis, and critical preoperative state), cardiac factors (unstable angina on intravenous nitrates, reduced left ventricular ejection fraction, recent myocardial infarction and pulmonary hypertension), and operation-related factors (emergency, major cardiac procedure other than isolated coronary surgery, thoracic aorta surgery and surgery for postinfarct septal rupture; Nashef et al., 1999). EuroSCORE has been utilized in quality control in cardiac surgery and offers risk stratification for the prediction of hospital mortality and the assessment of quality of care (De Maria et al., 2005).

Exclusion criteria were: intraoperative change of the surgical technique, surgical complications, or complications occurring in the ICU, emergency reoperation, renal failure, failure to agree to continue in the study, presence of other types of heart disease, and of pulmonary diseases.

The respiratory function of all patients was assessed by measuring MIP (cmH_2_O), MEP (cmH_2_O), and PEF (l/min) determined 1 day before the surgical procedure and during the period from the third to the seventh postoperative day. To evaluate maximal respiratory muscle force, the MIP and the MEP were measured with a digital pressure manometer WIKA-MV300 (WIKA Brasil Indústria Comércio, Iperó, Brazil), and PEF (l/min) determined with Assess peak flow meter (Philips Respironics). During the postoperative period in the ICU, the blood pressure of the patients was monitored at intervals of 30 min for 3 days and the mean arterial pressure (MAP) was thus obtained. MAP variability was determined on the basis of the standard deviation (SD), coefficient of variation (CV), variation independent of mean (VIM), average successive variability (ASV; Dolan and O'Brien, 2010).

The following time frame of outcome measures was followed:

1. preoperative: demographic, clinical EuroSCORE and respiratory measures (day-1)
2. intraoperative: volume of cardioplegia, CPB duration, aortic cross-clamp time, number of grafts
3. postoperative ICU (from surgery to 3rd day): blood pressure monitoring
4. postoperative (from 3rd to the 7th postoperative day): respiratory measures (day 3 to 7).

## Statistical analysis

Data were tested for normality using the D'Agostino & Pearson omnibus normality test. Data normally distributed are represented by mean (SE), and data that are not normally distributed are represented by median (interquartile range). Differences between different day-groups of respiratory parameters were examined using nonparametric test followed by Dunn's multiple comparisons against the group of the day before surgery (*p* < 0.05 was regarded as being statistically significant). Spearman's correlation coefficient r was used to quantify a relationship between two or more variables; a two-tailed *p*-value < 0.05 was considered statistically significant. Data were analyzed using statistical software (Prism 6 for Mac OS X).

## Results

### Demographic, euroscore, and intraoperative parameters

The study sample was composed of 44 patients with mean age of 62 ± 8.2 years. Demographic, clinical and surgical parameters were normally distributed within the study group (Table 1). None of the intraoperative parameters (cardioplegia volume, extracorporeal circulation time, aortic clamping time, number of grafts) was related to postoperative respiratory depression (data not shown).

**Table 1:** **Demographical, clinical, and intraoperative data for patients undergoing GABG**.

**VARIABLES**	
Age (years)	62 ± 8.2
Sex (Male/Female, %)	75%/25%
BMI (kg/m^2^)	26 [24–29]
Euroscore (%)	0.68 [0.54–0.77]
**COMORBIDITIES**	
Hypertension (%)	30.23
Diabetes (%)	6.98
Dyslipidaemia (%)	2.33
Hypertension + Diabetes (%)	16.28
Hypertension + Dyslipidaemia (%)	18.60
Hypertension + Diabetes + Dyslipidaemia (%)	2.33
Smoking (%)	6.82
**INTRAOPERATIVE VARIABLES**	
Volume of cardioplegia (ml)	1374 ± 440
CPB duration (min)	95 ± 29
Aortic cross-clamp time (min)	67 ± 27
Number of grafts (%)	
1	9.09
2	15.91
3	52.27
4	20.45
more than 4	2.27

*Data are presented as Mean ± SD, median [interquartile range]*.

### Respiratory parameters MIP, MEP and PEF are significantly depressed postoperatively

**Figure 1:**
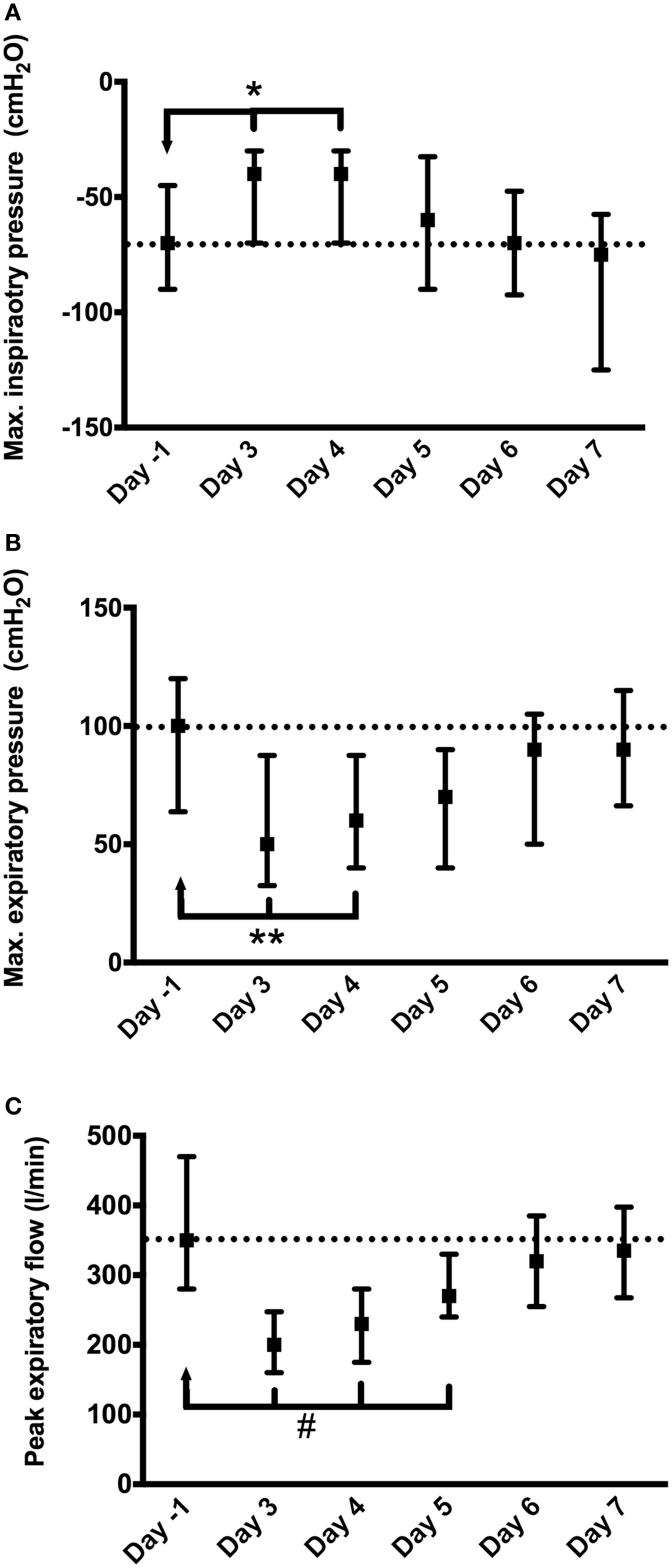
**Depression of respiratory function after CABG surgery: (A) maximal inspiratory pressure (MIP); (B) maximal expiratory pressure (MEP); (C) peak expiratory flow (PEF)**. Day-1, day before surgery; Day 3–7, days after surgery ICU (days 1–3 were at ICU). Statistical significance is in comparison to the day before surgery: ^*^p < 0.05; ^**^p < 0.01; #*p* < 0.001.

Respiratory parameters MIP, MEP, and PEF were assessed preoperatively (day-1) and from the third to the seventh postoperative day (post-ICU) (Figure 1). A marked reduction in lung function was present the first week after surgery, which was consistent with previous findings (Savci et al., 2011). Comparisons of respiratory parameters in the postoperative days after the ICU to those in the preoperative day revealed a significant depression on days 3–4 for MIPs (which reflects the strength of the diaphragm and other inspiratory muscles), days 3–4 for MEPs (which reflects the strength of the abdominal muscles and intercostal muscles) and days 3–5 for PEF (which is considered as a measure of pulmonary physiology and is being used as a surrogate for the more sensitive measurement of forced expiratory volume in 1 s; Slieker and van der Ent, 2003; Figure 1). As determined by MIP, MEP and PEF, the nadir of respiratory depression during the first week after cardiac surgery is the critical period when most of the respiratory complications occur (Albu et al., 2010).

### ICU blood pressure variability parameters associations with respiratory parameters

Of all the parameters of MAP variability, ASV showed a stronger positive and significant Spearman correlation (coefficients of 0.45–0.65, *p* < 0.01) with the aggravation of depression expressed by PEF up to the fifth day after heart surgery (Table 2).

**Table 2:** **Spearman's rank correlations (ρ) between the ICU MAP variability parameters (ASV, average successive variability; VIM, variation independent of mean; CV, coefficient of variation; SD, standard deviation) and respiratory parameters (PEF, peak expiratory flow; MEP, maximal expiratory pressure; MIP, maximal inspiratory pressure)**.

	**Day -1**	**Day 3**	**Day 4**	**Day 5**	**Day 6**	**Day 7**
	**PEF**	**PEF**	**PEF**	**PEF**	**PEF**	**PEF**
ASV	0.32	0.49^*^	0.56^*^	0.67^*^	0.53	0.30
VIM	−0.01	0.64^*^	0.46^*^	0.32	0.08	−0.50
CV	0.10	0.44^*^	0.48^*^	0.35	0.34	0.50
SD	0.15	0.36	0.42	0.32	0.33	0.50
	**MEP**	**MEP**	**MEP**	**MEP**	**MEP**	**MEP**
ASV	0.05	0.34	0.25	0.37	0.15	0.06
VIM	−0.18	0.49^*^	0.38	0.27	0.26	0.11
CV	−0.06	0.49^*^	0.55^*^	0.52^*^	0.39	−0.21
SD	0.00	0.39	0.49	0.46	0.30	0.32
	**MIP**	**MIP**	**MIP**	**MIP**	**MIP**	**MIP**
ASV	0.27	0.19	0.21	0.44	0.16	0.07
VIM	0.10	0.19	0.11	0.13	0.01	0.00
CV	0.16	0.15	0.27	0.20	−0.10	−0.60
SD	0.21	0.12	0.34	0.20	−0.14	−0.60

Gray shadowed cells correspond to the day when nadir of respiratory parameter depression occurs;

* *, significant Spearman's rank correlation p < 0.05*.

VIM MAP correlated with only the first day (of 2) of postoperative MEP nadir and the first 2 days (of 3) of postoperative PEF nadir (Table 2).

CV MAP was associated with the 2 days of postoperative MEP nadir but also with the 3rd day of MEP depression (Table 2). Also, CV MAP correlated with the first 2 days but not the 3rd day of postoperative PEF nadir.

SD of MAP at the ICU was not correlated with any respiratory parameter at any time (Table 2).

## Discussion

The main findings of this study are that the MAP variability monitored at ICU may have predictive value for the depression of respiratory function after ICU as determined by peak expiratory flow and maximal expiratory pressure. The ASV of ICU MAP correlated with the nadir of peak expiratory flow in all 3 days of depression in the week following ICU after cardiac surgery. The ICU blood pressure variability measures reflecting a sum-up of the perioperative measures producing neurohumoral challenges may predict respiratory outcomes after cardiac surgery.

The pathophysiological determinants of the cardiac surgery-induced cardiovascular and respiratory dysfunction are subject of actual research and comprise a complex combination of intraoperative factors such as general anesthesia (Hachenberg et al., 1993), surgical injury caused by sternotomy (Berrizbeitia et al., 1989), damage of phrenic nerve (Mok et al., 1991), mechanical ventilation (Roosens et al., 2002), cardiopulmonary bypass (Babik et al., 2003), and postoperative airway narrowing (Babik et al., 2003; Albu et al., 2010). Cardiac surgery-induced respiratory function can be afflicted not only directly through mechanisms described above but also through systemic reactions including sympathetic stress response, pain (Ledowski et al., 2012), and inflammation (Paparella et al., 2002).

Cardiovascular and respiratory systems have vital functions in the general adaptation to stress, and both are coordinated by the autonomic nervous system. Surgery-associated stress induces a dysautonomia that afflicts cardiovascular and respiratory functions (Garcia et al., 2013). Cardiovascular and respiratory activities are intricately coupled through highly overlapping brainstem autonomic control centers. This cardiorespiratory coupling involves multiple mechanisms mediating the bidirectional influences between the cardiovascular and respiratory activity (Dick et al., 2014). The intraoperative blood pressure variability is a strong predictor of 30-day postoperative mortality after CABG surgery (Aronson et al., 2010). On the other hand, inspiratory muscle fatigue may increase sympathetic vasomotor outflow (Katayama et al., 2012). These interrelations may contribute to the pathophysiology of cardiorespiratory coupling, and indicate a possible bidirectional connection between alterations of respiratory function and blood pressure variability. Indeed, our study suggests that MAP variability assessment during ICU, which may reflect the autonomic and neurohumoral reaction to perioperative stress, is a good predictor of the nadir of respiratory depression that occurs in the first postoperative week of CABG surgery. These complex mechanisms involved in the surgery-associated stress induced by composite intraoperative factors affecting autonomic nervous system and cardiorespiratory coupling may explain the predictive nature between cardiovascular and respiratory functions observed in our study.

Predictive biomarkers should be considered to recognize and manage postoperative complications (Gonzalez et al., 2014). Perioperative assessment of surgery is important in order to minimize the surgical risks and to prevent postoperative complications (Task Force et al., 2013). Our findings may indicate ICU MAP variability as a measure in order to initiate inspiratory muscle training programs (Westerdahl et al., 2005; Savci et al., 2011) or physiotherapy programs (Stein et al., 2009; Hirschhorn et al., 2012) for faster recovery and improved pulmonary function after CABG.

In summary, our study evidenced a predictive value of ICU MAP variability on postoperative respiratory depression. Further studies may uncover if there may be additional benefit in also reducing MAP variability to prevent postoperative cardiovascular events (Dolan and O'Brien, 2010).

Some limitations must be considered.

Since we aimed at studying patients without confounding effects of postoperative complications on respiratory function, we had enrolled a cohort without intraoperative complications and of low risk EuroSCORE (European System for Cardiac Operative Risk Evaluation) with < 2. Therefore, there were no prolonged stay of intensive care and no postoperative complications were observed in the investigated period. Nevertheless, the timeframe of the investigation did not allow further monitoring in order to observe influences on clinical outcomes. Thus, we don't know if the respiratory depression further translated or not in clinical events.

Another limitation is the small sample size of the study. Also, since exclusion criteria were very strict, the results cannot be generalized to a broader range of patients such as the ones with perioperative complications. This strict selection process was intended to control several biases that may otherwise have limited the validity of the analysis. Our results are likely to be transferable to cardiac surgery patients without perioperative complications and further studies with broader inclusion criteria and longer timeframe of investigations are necessary.

## Author contributions

AC carried out the postoperative patient care including the respiratory function evaluations and perioperative data collection, and participated in the design of the study. PC carried out the cardiac surgery and patient selection. CD carried out the cardiac surgery and patient selection. LP participated in the statistical analysis. LC participated in the design of the study and coordination, statistical analysis, and contributed to the interpretation of the results and the drafting of the manuscript. OB conceived the study, and participated in its design and coordination and drafted the manuscript.

## Funding

This work was supported by the São Paulo Research Foundation (grant numbers FAPESP 2013/14724-0, FAPESP 13/06698-0). OB is supported by the National Council for Scientific and Technological Development (CNPq, 301706/2013-1).

### Conflict of interest statement

The authors declare that the research was conducted in the absence of any commercial or financial relationships that could be construed as a potential conflict of interest.

## Abbreviations

CABG: coronary artery bypass surgery
CPB: with cardiopulmonary bypass
ICU: intensive care unit
MIP: maximal inspiratory pressure
MEP: maximal expiratory pressure
PEF: peak expiratory flow
MAP: mean arterial pressure
SD: standard deviation
CV: coefficient of variation
VIM: variation independent of mean
ASV: average successive variability.
